# Non-Healing Wound Model in Diabetic C57BL/6 Mice

**DOI:** 10.3390/mps9040104

**Published:** 2026-07-03

**Authors:** Lyubov A. Rzhanova, Ekaterina V. Kuzmenko, Alena A. Permyakova, Andrei A. Riabinin, Evgenii S. Ruchko, Maria B. Chernysheva, Ekaterina A. Vorotelyak, Elena I. Morgun

**Affiliations:** 1 Koltzov Institute of Developmental Biology of the Russian Academy of Sciences, 119334 Moscow, Russia; katya.3@bk.ru (E.V.K.); dex.winner@gmail.com (A.A.P.); andrey951233@mail.ru (A.A.R.); ruchkoevgeny@yandex.ru (E.S.R.); m.b.cher@gmail.com (M.B.C.); 2 Moscow Institute of Physics and Technology, 141701 Dolgoprudny, Russia; 3 Biological Faculty, Lomonosov Moscow State University, 119234 Moscow, Russia; 4 Department of Histology, Cytologyand Embryology, People’s Friendship University of Russia Named After Patrice Lumumba (RUDN University), 117198 Moscow, Russia

**Keywords:** regeneration, non-healing wound, diabetes

## Abstract

**Background**: This study focuses on developing a model of a non-healing wound that recapitulates the pathogenesis of the corresponding human pathology. **Methods**: A non-healing wound was modeled in mice with streptozotocin-induced diabetes. The following parameters were assessed: re-epithelialization, epidermal hypertrophy, wound contraction, relief index, angiogenesis, and granulation tissue maturation. These parameters were compared between diabetic mice and healthy controls. **Results**: The proposed model demonstrated a significant delay in regenerative processes compared to healthy animals. **Conclusions**: These findings **support the relevance** of this model to human pathology and **indicate that it may be applicable** for preclinical studies of drugs aimed at promoting wound regeneration.

## 1. Introduction

The study of tissue regeneration, particularly of the skin, represents a significant challenge in modern biomedicine. Under physiological conditions, the repair of a skin wound culminates in the complete restoration of the organ’s structure and function. However, in the presence of complicating factors such as infection, hypoxia, or immune dysfunction, the wound healing process can become chronic [1]. The pathogenesis of non-healing wounds is characterized by persistent inflammation, elevated proteolytic activity, and inhibited synthesis of extracellular matrix components [2]. Key risk factors include diabetes mellitus, vascular insufficiency, cachexia, advanced age, and local infection [3,4]. Clinically, these factors manifest as pressure sores, diabetic ulcers, and ischemic ulcers, all of which are notoriously resistant to standard therapy.

In this context, the development of adequate experimental models that faithfully recapitulate the key features of human pathology is of paramount importance. It is well established that the unique structural characteristics of laboratory animal skin present significant challenges in modeling chronic ischemic wounds [5]. While the common splinting method in rodents effectively prevents wound contraction and prolongs healing time, it fails to reproduce the ischemic nature of the pathology, and the presence of the synthetic ring itself constitutes a significant artifact. Our laboratory has previously introduced a model of an ischemic non-healing wound that is relevant to human pathology and suitable for preclinical studies [6,7]. However, this model possesses certain drawbacks, including the artifactual influence of sutures placed near the wound and the risk of bleeding during vascular ligation. The latter can lead to animal mortality and confound the interpretation of experimental results.

A review of the literature reveals several existing models of diabetic wounds, each with substantial limitations. In one study [8], a wound of only 1 mm in diameter was created on the ears of mice with alloxan-induced diabetes, a size that is extremely inconvenient for dynamic monitoring and preclinical testing of cell-containing products (e.g., skin equivalents). Other studies have used standard circular wounds of 6 mm in diameter on db/db mice or mice with streptozotocin-induced diabetes [9,10]. However, a major limitation associated with modeling skin wounds in mice is that, unlike in humans, wound contraction mediated by the *panniculus carnosus* muscle contributes significantly to wound closure.

To minimize the contribution of contraction, we selected a square wound shape. Mawaki et al. (2007) [11] demonstrated that although the overall dynamics of area reduction are similar for square and circular wounds in mice, the square shape creates a heterogeneous myofibroblast activity profile: early loss of myofibroblasts at the wound corners prevents their constriction. In the context of diabetes, which itself impairs myofibroblast function and differentiation, this spatial imbalance in contraction may further delay remodeling and promote the formation of a stellate scar. Therefore, a square wound shape was chosen for our model.

Wound size is also a critical parameter. The rate of skin regeneration in mice is very high: a wound 5–7 mm in diameter in a healthy animal heals completely within 14 days, which complicates dynamic studies of regeneration, including those testing drugs and tissue-engineered products. Conversely, excessively large wounds in mice, unlike in humans, heal through epimorphic regeneration via the induction of wound-induced hair follicle neogenesis (WIHN). The initiation of this process depends on a critical wound size threshold. Specifically, reliable WIHN induction requires wounds of 1.5 × 1.5 cm in mice [12]. Since our goal was to create a wound sufficiently large for dynamic monitoring and product testing but not large enough to trigger alternative regenerative mechanisms (WIHN), a size of 1 × 1 cm was chosen as optimal.

Thus, based on the need to: (1) use diabetic animals, (2) minimize wound contraction (square shape), and (3) select a size sufficient for dynamic studies but not inducing WIHN, the available literature lacks studies that combine all these selected parameters (streptozotocin-induced diabetes, mice, square 1 × 1 cm wound) within a single validated model.

**Aim of the study.** To develop and validate a protocol for a non-healing diabetic wound model in mice using a square wound of 1 cm^2^.

## 2. Materials and Methods

### 2.1. Induction of Diabetes

Experiments were conducted on C57BL/6 male four-week-old mice with an average body weight of 20–22 g. The animals were housed under standard conditions in the vivarium of the Institute of Developmental Biology of the Russian Academy of Sciences (IDB RAS), in groups of 4–6 mice per cage, with ad libitum access to food and water. Animal care was supervised by the vivarium staff, and compliance with quarantine and sanitary regulations was monitored by the attending veterinarian. All experimental procedures were approved by the IDB RAS Institutional Animal Care and Use Committee (Protocol № 74, of September 2023 and Protocol № 88 of December 2024).

The diabetes model was established by administering streptozotocin (Sigma-Aldrich, Taufkirchen, Germany) five times at a dose of 50 mg/kg body weight, dissolved in saline solution, with an injection volume of 100–140 µL (4.8 mL/kg body weight). Injections were performed daily from days 1 to 5 of the experiment.

Blood samples for glucose measurement were collected from the tail vein every 48 h, starting from day 7 of the experiment. Blood glucose concentrations were determined using a Contour TS portable glucometer (Bayer, Leverkusen, Germany).

Following the measurements on days 10 and 12, animals exhibiting severe hyperglycemia (blood glucose levels exceeding 15 mmol/L) were selected as high responders.

### 2.2. Development of a Non-Healing Wound Model

The study used 16 mice from the control group and 16 mice from the diabetic group (total sample size *n* = 32). All animals were assigned to two experimental groups: diabetic and control. Each experimental group comprised 5–6 mice (Table 1).

A full-thickness excisional wound was created in the interscapular region. The wound was square-shaped, with each side measuring 1 cm (area of 1 cm^2^) and a depth of 3 mm. The wounds were subsequently covered with a transparent adhesive dressing (Tegaderm, Maplewood, MN, USA).

Animals were euthanized on days 5, 10, and 15 post-wounding by anesthetic overdose, followed by tissue sample collection for analysis.

All animal procedures were performed under general anesthesia.

### 2.3. Tissue Processing and Histology

Wound tissue was divided into equal segments for subsequent histological and immunohistochemical analysis. Tissue fragments were embedded in O.C.T. compound (Sakura, Tokyo, Japan, catalog no. 4583), frozen, and stored at −80 °C. Sections (10 μm) were cut using a cryostat Leica CM1950 (Wetzlar, Germany), fixed in 4% paraformaldehyde, stained with hematoxylin (BioVitrum, catalog no. 05-001, Saint Petersburg, Russia) and eosin, and mounted with BioMount synthetic medium (Bio-Optica, Milano, Italy). Micrographs were obtained using a Keyence BZ-9000 microscope (Osaka, Japan).

### 2.4. Immunofluorescence Staining

Sections were fixed in 4% PFA (P6148-500G, Sigma-Aldrich, St. Louis, MO, USA) for 20 min, washed with PBS, and blocked with 5% bovine serum albumin. Staining with primary antibodies against CD31 (ab56299, Abcam, Cambridge, UK; 1:100) and CK14 (ab181595, Abcam; 1:500) was performed overnight at 4 °C. After washing, sections were incubated with appropriate secondary antibodies (1:1000) for 1 h at room temperature, followed by DAPI (1:5000) counterstaining. Antibody-stained sections were visualized by fluorescence microscopy on a Leica DMI6000 microscope (Wetzlar, Germany). The exposure time was 11.5 ms for the red channel, 17.257 ms for the green channel, and 11.5 ms for the blue channel.

### 2.5. Morphometric Analysis

Morphometric analysis was performed using ImageJ 1.54g (National Institutes of Health, USA) software. Histological sections stained with hematoxylin and eosin (H&E) were analyzed to assess re-epithelialization, wound contraction, wound closure, mean number of epidermal layers at the wound margins, relief index, and stratification of granulation tissue layers.

In the morphometric analysis, day 0 refers to the wound immediately following surgery, specifically a square wound with an area of 1 cm^2^ (i.e., 1 cm × 1 cm).

Angiogenesis in the wound bed was evaluated based on vessel area, percentage of vessel area relative to the total wound bed area, and the distribution of short (≤30 μm) and long (>30 μm) vessels in sections immunostained with antibodies against the endothelial marker CD31.

### 2.6. Statistical Analysis

Statistical analysis was performed using GraphPad Prism version 8.0 for Windows (GraphPad Software, San Diego, CA, USA) software. Data were analyzed using the nonparametric Kruskal–Wallis test followed by Dunn’s post hoc correction for multiple comparisons. Statistical significance was denoted as * *p* < 0.05, ** *p* < 0.01, *** *p* < 0.001, and **** *p* < 0.0001.

## 3. Results and Discussion

The dynamics of regeneration in acute and non-healing wound models were assessed using the following parameters: wound bed area, re-epithelialization, number of cell layers in the epidermal tongues at the wound margins, wound contraction, wound closure, relief index, angiogenesis, and wound bed layer ratio.

**Figure 1:**
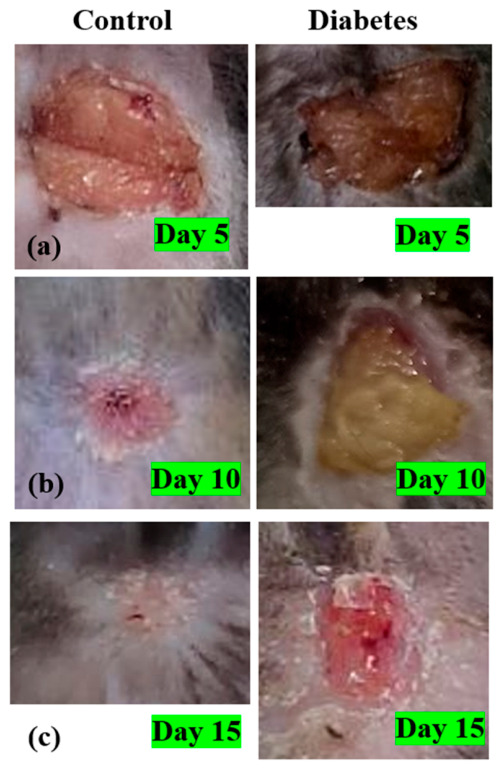
Macroscopic images of wounds from the control and diabetic groups on days 5 (**a**), 10 (**b**), and 15 (**c**).

The general appearance of wounds in mice from both groups is presented in Figure 1.

### 3.1. Stratification of Granulation Tissue Layers Within the Wound Bed

**Figure 2:**
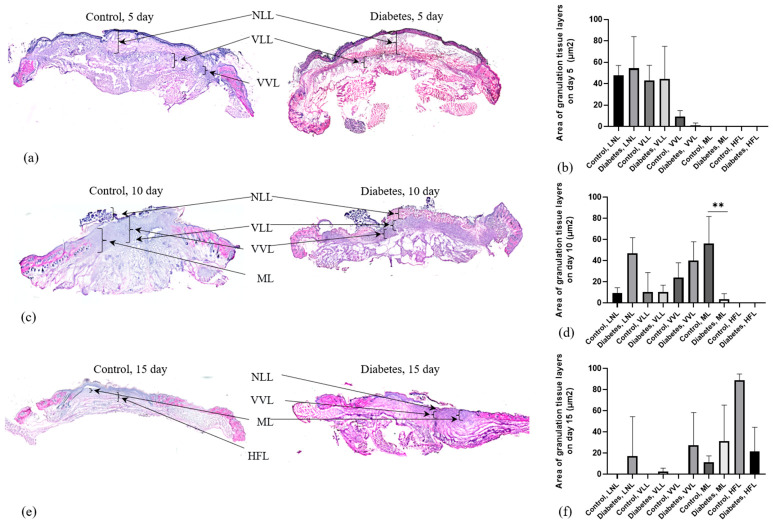
Comparative analysis of granulation tissue stratification in control and diabetic mice. Structural organization and quantitative distribution of tissue layers: (**a**,**c**,**e**) Histological organization of granulation tissue. Representative sections showing layer stratification on (**a**) day 5, (**c**) day 10, and (**e**) day 15. (**b**,**d**,**f**) Proportion of granulation tissue layers. Grouped bar charts showing the percentage distribution of tissue layers in the wound bed on (**b**) day 5, (**d**) day 10, and (**f**) day 15. Key to tissue layers: LNL: Superficial leukocyte-necrotic layer; VLL: Vascular loop layer; VVL: Vertical vessel layer; ML: Maturing layer; HFL: Horizontal fibroblast layer. Statistics: Statistical analysis was performed using the non-parametric Kruskal–Wallis test followed by Dunn’s post hoc test for multiple comparisons. Data are presented as median with interquartile range (or mean ± SD). ** *p* < 0.01.

According to the classical morphological paradigm [13], the maturation of granulation tissue proceeds through a sequential progression of distinct layers: the superficial leukocyte-necrotic layer (LNL), the vascular loop layer (VLL), the vertical vessel layer (VVL), the maturing layer (ML), and the horizontal fibroblast layer (HVL). To elucidate the impact of diabetes on this spatiotemporal sequence, we performed a comparative morphometric analysis of the relative areas occupied by these key layers on post-operative days 5, 10, and 15 (Figure 2a,c,e).

The area of a specific histological layer was evaluated in accordance with the formula:

$$P_{layer}=\frac{S_{layer}}{S_{total\_gt}}\times 100\%$$

S_layer_—area of a specific histological layer (LNL, VVL, VLL, ML, or HVL), µm^2^,
S_total_gt_—total area of granulation tissue within the wound bed on the section, µm^2^.

On day 5, granulation tissue in both groups was predominantly composed of immature constituents, indicating that the wounds were at the inflammatory stage (Figure 2a). The control group exhibited features consistent with active inflammation and the onset of proliferation: the LNL constituted 47.85 ± 9.21% of the wound bed, while the VLL comprised 42.76 ± 14.35%. The VVL was in its nascent stage, representing only 9.39 ± 5.46% of the area. The relatively low standard deviation observed in controls indicated a uniform wound healing response among animals in this group (Figure 2b).

In the diabetic group, the mean proportional areas of the LNL (54.29 ± 29.70%) and VVL (44.56 ± 30.27%) were statistically comparable to the control group. However, the markedly elevated standard deviations for these parameters revealed pronounced inter-individual heterogeneity in the wound status from the earliest time point. This variability ranged from minimal inflammation (LNL = 4.15%) in some animals to extensive necrosis (LNL = 80.16%) in others (Figure 2b).

By day 10, the wounds of both groups were in the proliferation phase (Figure 2c). However, wound morphology differed significantly between the control and diabetic groups. In the control group, the relative area of the LNL decreased, and the ML emerged as the dominant compartment. Conversely, the diabetic group exhibited a feature of protracted inflammation: the proportional area of the LNL remained elevated and was higher than in the control group. However, these changes did not reach statistical significance. Concomitantly, the proportion of the ML was statistically significantly reduced in the diabetic cohort relative to the control group (Figure 2d), indicative of impaired tissue maturation. This delay was further substantiated by more pronounced infiltration of polymorphonuclear leukocytes into the deep layers of granulation tissue in diabetic animals.

By day 15, the healing process in the control group had reached the terminal stages of remodeling and re-epithelization (Figure 2e). Granulation tissue was almost entirely transformed into a mature fibrous layer (FL), which accounted for 88.71 ± 6.03% of the wound bed. Immature layers (LNL, VVL, VLL) were completely absent. However, granulation tissue remodeling in the diabetic group remained incomplete. Specifically, the proportion of the HFL was lower in the diabetic group compared with controls, while the proportion of the VVL remained elevated (Figure 2f). These values did not reach statistical significance.

Thus, morphometric analysis of granulation tissue architecture demonstrates that, in the proposed model, diabetes mellitus does not simply delay morphogenetic progression in the wound bed. Instead, it drives a combination of prolonged inflammatory processes that impede the transition to the phases of remodelling and re-epithelization.

### 3.2. Wound Bed Area

**Figure 3:**
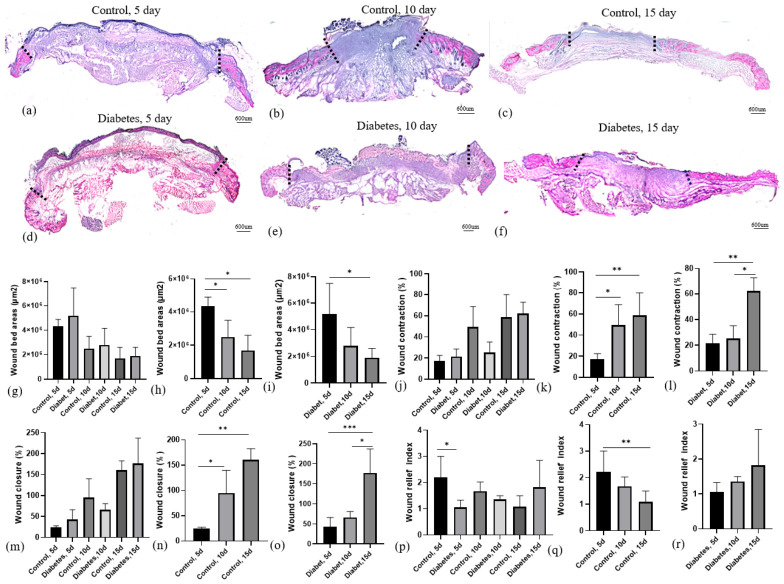
Histological and morphometric assessment of wound healing in control and diabetic mice. General view and quantitative analysis of skin repair dynamics: (**a**–**f**) Histological sections of the wound area. Microphotographs of mouse skin stained with hematoxylin and eosin (H&E), magnification ×40. Black dashed lines indicate the boundaries of the wound bed. (**g**–**i**) Wound bed area dynamics. Grouped bar charts showing: (**g**) comparative analysis between control and diabetes groups; (**h**) dynamics in the control group; (**i**) dynamics in the diabetes group. (**j**–**l**) Wound contraction. Grouped bar charts showing: (**j**) comparative analysis between groups; (**k**) dynamics in the control group; (**l**) dynamics in the diabetes group. (**m**–**o**) Wound closure. Grouped bar charts showing: (**m**) comparative analysis between groups; (**n**) dynamics in the control group; (**o**) dynamics in the diabetes group. (**p**–**r**) Wound relief index. Grouped bar charts showing: (**p**) comparative analysis between groups; (**q**) dynamics in the control group; (**r**) dynamics in the diabetes group. Statistics: Statistical analysis was performed using the non-parametric Kruskal–Wallis test followed by Dunn’s post hoc test for multiple comparisons. Data are presented as median with interquartile range (or mean ± SD). * *p* < 0.05, ** *p* < 0.01 *** *p* < 0.001.

Quantitative analysis of the wound bed area revealed that on postoperative day 5, it measured 4.33 ± 0.55 × 10^6^ µm^2^ in the control group and 5.18 ± 2.31 × 10^6^ µm^2^ in the diabetes group (Figure 3a–f). By day 10, the wound area had significantly decreased in both groups, reaching minimal values by day 15. No statistically significant differences were observed between the control and diabetes groups at any of the time points examined (Figure 3g). Moreover, both groups exhibited the expected temporal dynamics, with a significant reduction in wound area occurring between days 5 and 10, as well as between days 5 and 15 (Figure 3h,i). These findings demonstrate that the overall dynamics of wound area reduction are preserved under diabetic conditions.

### 3.3. Wound Bed Contraction

Wound contraction, defined as the reduction in size of a cutaneous defect during regeneration, is mediated by myofibroblasts and, in rodents, the striated panniculus carnosus muscle. The wound contraction was evaluated in accordance with the formula:

$$W_{c}=\frac{L_{o}-L_{t}}{L_{0}}\times 100\%$$

L_0_—initial wound defect width (on day 0), µm.
L_t_—mean width of the wound bed on a given day of the experiment (t), µm.

Statistical analysis revealed no significant difference in the percentage of wound contraction between the control and diabetes groups (Figure 3j).

Intragroup dynamics differed markedly over time. In the control group, a significant increase in contraction occurred between days 5 and 10, after which values stabilized, with no significant change between days 10 and 15 (Figure 3k). In contrast, the diabetic group exhibited no significant increase in contraction during the early phase (days 5–10). A significant increase was observed only during the later interval (days 10–15), by which time contraction levels had reached those of the control group (Figure 3l).

These findings indicate that the principal effect of diabetes on wound contraction is a pronounced temporal delay. This is likely attributable to myofibroblast dysfunction under diabetic conditions, potentially involving alterations in timely differentiation, contractile activity, or spatial coordination within granulation tissue.

### 3.4. Wound Closure

Wound closure was assessed as an integrative parameter reflecting two key processes: re-epithelialization of the wound surface and contraction of the wound bed. Wound closure was evaluated in accordance with the formula:

$$W_{cl}\;={\;W}_{c}\;+\;R_{int}$$

W_c_—Wound Contraction
R_int_—Re-epithelialization, Integral Method

Statistical analysis revealed no significant differences in wound closure values between the control and diabetes groups on days 5, 10, or 15 (Figure 3m). However, the temporal dynamics within each group differed markedly.

In the control group, a significant increase in the percentage of wound closure was observed between days 5 and 10, as well as between days 5 and 15, indicating continuous and progressive healing (Figure 3n). In contrast, the diabetic group exhibited no significant change during the early phase (days 5–10), with the principal increase occurring between days 10 and 15 (Figure 3o).

Thus, although the cumulative rate of wound closure by day 15 did not differ statistically between the diabetic and control groups, the underlying healing process was qualitatively distinct. The diabetic group was characterized by disrupted dynamics: a critical delay in contraction—marked by a shift in the peak activity from day 10 to day 15—was accompanied by incomplete and variable re-epithelialization. Consequently, by the end of the experimental period, wounds in the diabetic group, despite a high percentage of closure, remained morphologically immature, exhibiting features such as incomplete epithelialization and hypertrophy of the marginal epidermis. These alterations recapitulate key characteristics of chronic wound pathology observed in human diabetic conditions.

### 3.5. Relief Index

To assess the maturity and thickness of the developing granulation tissue, the “relief index” was employed. This parameter was defined as the ratio of the thickness of the granulation layer within the wound bed to the thickness of the adjacent intact dermis.

Relief index was evaluated in accordance with the formula:

$$I_{r}=\frac{H_{gt}}{H_{id}}$$

H_gt_—mean thickness of the granulation tissue layer within the wound bed, µm.
H_id_—mean thickness of the adjacent intact dermis, µm.

Quantitative analysis revealed that on day 5, the relief index was significantly higher in the control group compared to the diabetic group. On days 10 and 15, no statistically significant intergroup differences were observed (Figure 3p). Intragroup dynamics of the parameter also exhibited distinct patterns. In the control group, a significant decrease in the relief index was noted by day 15 relative to day 5, reflecting the normal physiological process of granulation tissue maturation and subsequent flattening (Figure 3q). Conversely, the diabetic group showed no significant dynamic changes across the observed time points; however, a tendency towards an increase in the index was observed by day 15 (Figure 3r). In conclusion, the early stages of regeneration in the diabetic group are characterized by relatively thinner granulation tissue, as indicated by a lower relief index. This finding is suggestive of impaired granulation tissue formation, which is typically associated with chronic, non-healing wounds. Furthermore, diabetes mellitus was associated with disrupted granulation tissue remodeling dynamics: while the control group demonstrated the expected decrease in the relative thickness of the granulation layer by day 15, indicative of proper wound maturation, this remodeling process was impaired under diabetic conditions.

### 3.6. Re-Epithelization

To quantify wound defect healing, three methods were employed, each differing in the normalization approach for the measured length of the epithelial tongue and, consequently, in the biological interpretation of the resulting parameters.

The **integral method** provides a cumulative estimate of the proportion of the original defect covered by the epithelium at a given time point, thereby reflecting overall healing progress. The integral method for quantifying re-epithelialization was evaluated in accordance with the formula:

$$R_{int}=\frac{l_{left}+l_{right}}{L_{0}}\times 100\%$$

l_left_, l_right_—length of the left and right marginal epidermis on a given day of the experiment (t), µm.
L_0_—initial wound width on day 0, µm.

The **interval rate** allows assessment of the intensity of epithelialization at each stage while minimizing the confounding effect of prior wound contraction. The interval rate of re-epithelialization was calculated in accordance with the formula:

$$R_{rate}=\frac{l_{left}+l_{right}}{L_{t0}}\times 100\%$$

l_left_, l_right_—length of the left and right marginal epidermis on a given day of the experiment (t), µm.
L_t0_—initial wound width at the beginning of the interval: day 0 for the 0–5 day interval, day 5 for the 5–10 day interval, and day 10 for the 10–15 day interval, µm.

The **proportion of the current defect** characterizes the efficiency of epithelialization under dynamically changing wound area conditions. This parameter was evaluated in accordance with the formula:

$$R_{curr}=\frac{l_{left}+l_{right}}{L_{t}}\times 100\%$$

l_left_, l_right_—length of the left and right marginal epidermis on a given day of the experiment (t), µm.
L_t_—actual width of the wound bed between the edges of the injured dermis on the same day (t), µm.

The combined application of these three approaches enables the dissociation of the relative contributions of epithelialization and wound contraction, as well as the identification of hidden impairments in the reparative process.

**Integral method**

This parameter was calculated as the percentage ratio of the length of the marginal epidermis at the study time points (days 5, 10, and 15) to the initial wound defect width (day 0). Thus, the indicator reflects the proportion of the original defect closed by newly formed epithelium by a given date, characterizing the integral outcome of epithelialization over the entire preceding period. Because the denominator remains fixed, this method does not allow the contributions of epithelialization and marginal contraction to be dissociated; nevertheless, it provides an overall assessment of wound closure progress.

**Figure 4:**
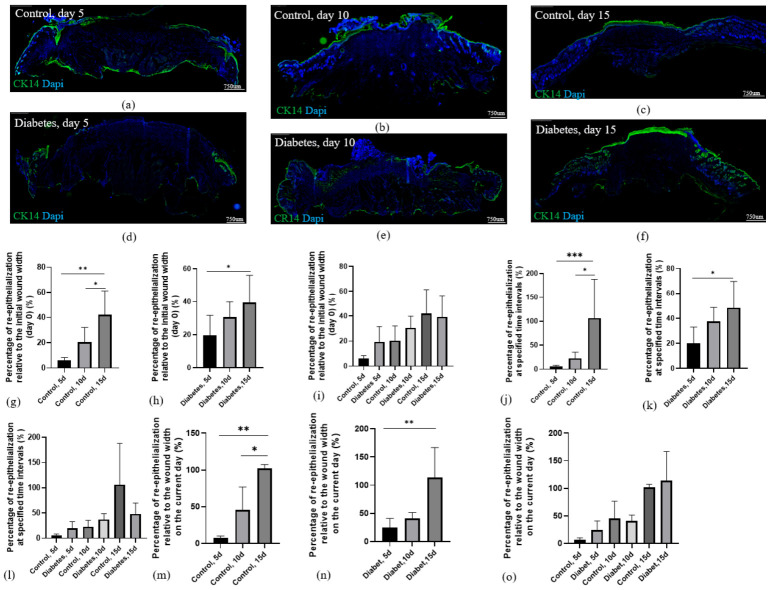
Re-epithelialization dynamics of wound defects in control and diabetic mice. Immunohistochemical assessment and quantitative analysis of epithelial repair: (**a**–**f**) CK14 staining of wound sections. Microphotographs showing Cytokeratin 14 (CK14) expression in the regenerating epithelium. (**g**–**i**) Percentage of re-epithelialization. Grouped bar charts showing progress relative to the initial wound width (day 0): (**g**) control group; (**h**) diabetes group; (**i**) comparative analysis between groups. (**j**–**l**) Marginal epithelialization rate. Dynamics during sequential healing phases (intervals: 0–5, 5–10, and 10–15 days): (**j**) control group; (**k**) diabetes group; (**l**) comparative analysis between groups at all time points. (**m**–**o**) Current wound defect re-epithelialization ratio. Dynamics on days 5, 10, and 15: (**m**) control group; (**n**) diabetes group; (**o**) comparative analysis between groups. Statistics: Statistical analysis was performed using the non-parametric Kruskal–Wallis test followed by Dunn’s post hoc test for multiple comparisons. Data are presented as median with interquartile range (or mean ± SD). * *p* < 0.05, ** *p* < 0.01 *** *p* < 0.001.

In the control group, progressive dynamics of accumulated epithelialization were observed. Intragroup analysis revealed a statistically significant increase between days 15 and 5, as well as between days 15 and 10 (Figure 4g). The lack of significant differences between days 5 and 10 indicates a relatively slow accumulation of epithelialization during the first 10 days, typical of the preparatory phase, after which the process accelerates.

In contrast, the group with simulated diabetes mellitus exhibited different dynamics of accumulated epithelialization. Intragroup analysis demonstrated a lack of stable progression: a statistically significant increase was recorded only when comparing the extreme time points (days 5 vs. 15). The differences between adjacent time points (days 5 vs. 10 and days 10 vs. 15) did not reach statistical significance (Figure 4h). This finding points to an uneven accumulation of epithelialization and the absence of a distinct phase of active defect closure at later stages.

Direct intergroup comparisons at the corresponding follow-up time points revealed no statistically significant differences (Figure 4i).

**Marginal epithelialization rate**

To assess the intensity of epithelialization at each healing stage, the rate of marginal epithelialization was analyzed. This parameter was calculated as the percentage ratio of the length of newly formed epidermis over a given interval to the mean wound defect width at the beginning of that interval: for interval 0–5 days—to the initial wound width (day 0); for interval 5–10 days—to the mean wound width on day 5; for interval 10–15 days—to the mean wound width on day 10. Normalization to the wound width at the start of each interval minimizes the confounding effect of prior marginal contraction, thereby enabling a direct assessment of keratinocyte migration activity at each stage. Consequently, this method provides insight into the dynamics of the pure epithelialization rate.

In the control group, intragroup statistical analysis revealed significant differences between intervals 0–5 and 10–15 days, as well as between 5–10 and 10–15 days (Figure 4j). In contrast, the diabetic group showed a statistically significant increase in the rate only when comparing the extreme intervals (10–15 vs. 0–5 days). Differences between adjacent intervals did not reach statistical significance (Figure 4k). This finding indicates the absence of the proliferative peak characteristic of normal healing at late stages and suggests a “smoothened” pattern of the reparative process under diabetic conditions.

Intergroup comparison revealed no statistically significant differences (Figure 4l). Overall, these results confirm the trends observed with the integral method, namely, a delay in re-epithelialization processes in diabetic wounds during the proliferative stage (by day 10).

**Proportion of the current defect**

To assess the effectiveness of epithelialization under conditions of dynamically changing wound area, the parameter “proportion of the current defect” was calculated. This indicator reflects the percentage ratio of the length of the newly formed epidermis to the actual width of the wound bed on the same day.

In the control group, a steady increase in the proportion of the current defect closure was observed, reflecting the progressive development of the reparative process. Intra-group statistical analysis confirmed the high significance of all dynamic stages, with differences between all time points reaching statistical significance. These findings indicate a stable and progressive nature of epithelialization throughout the observation period in the control group (Figure 4m).

In contrast, the diabetic group exhibited a different pattern of dynamics, characterized by high variability and irregularity of the process. Differences between days 5 and 10 did not reach statistical significance. Notably, an extremely high standard deviation was observed on day 15, indicating a markedly heterogeneous response among animals in the diabetic group: some individuals showed complete wound closure with epithelial hyperplasia, while others exhibited a significant delay in epithelialization (Figure 4n).

Intergroup comparison revealed no statistically significant differences at any of the follow-up time points (Figure 4o). Analysis of the proportion of the current defect thus enabled evaluation of epithelialization efficacy under changing wound geometry and helped identify important pathophysiological features.

In conclusion, the evaluation of re-epithelialization using all three methods indicates an impairment of the dynamics of the reparative process during diabetic wound regeneration in the proposed model.

### 3.7. Stratification of Proliferating Epidermal Tips at the Wound Edge

**Figure 5:**
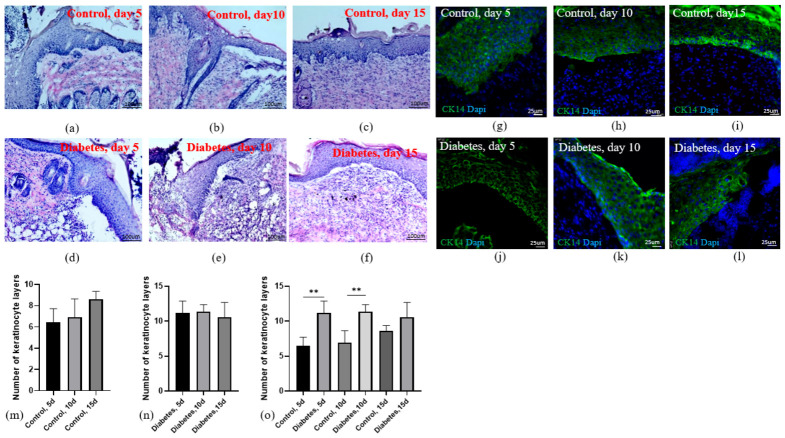
Assessment of the leading edge and epidermal tip proliferation. Morphological analysis and quantification of epidermal ridge formation: (**a**–**f**) Histological sections of the wound leading edge. Microphotographs of mouse skin stained with hematoxylin and eosin (H&E), magnification ×40. (**g**–**l**) Immunohistochemical detection of CK14. High-magnification (×400) microphotographs showing Cytokeratin 14 expression at the epidermal tips. (**m**–**o**) Epidermal tip proliferation (cell layers). Grouped bar charts showing the number of cell layers in the epidermal ridges: (m) dynamics in the control group; (**n**) dynamics in the diabetes group; (**o**) comparative analysis between groups on days 5, 10, and 15. Statistics: Statistical analysis was performed using the non-parametric Kruskal–Wallis test followed by Dunn’s post hoc test for multiple comparisons. Data are presented as median with interquartile range (or mean ± SD). ** *p* < 0.01.

During the healing of a full-thickness skin defect, gradual re-epithelialization occurs, accompanied by hyperproliferation of the newly formed epidermis at the wound edges, leading to the formation of epidermal tongues (Figure 5a–l). In mice, under normal conditions, the number of cell layers in the marginal epidermis subsequently reduces to 2–3 rows upon completion of healing. To assess the degree of epidermal hypertrophy and compare the rate of skin defect regeneration between the control and diabetic groups, the number of cell layers within the marginal epidermis was quantified. Statistical analysis revealed marked intergroup differences. On day 5 post-wounding, the number of epithelial layers in the diabetic group was significantly higher compared to the control group. This difference persisted through day 10 (Figure 5o). By day 15, the thickness of the epidermal tongues in the control group had decreased to 8.64 ± 0.73 layers, whereas in the diabetic group it remained elevated at 10.60 ± 2.08 layers. Although the difference between groups did not reach statistical significance at this final time point, a clear trend is evident in the diagram (Figure 5o).

Thus, the diabetic condition is characterized by persistent hypertrophy of the marginal epidermis, manifesting as early as day 5 and sustained throughout the observation period. The absence of a reduction in the number of keratinocyte layers by day 15 in the diabetic group indicates a delay in the completion of the active proliferation phase and points to impaired maturation and remodeling of the newly formed epithelium.

### 3.8. Angiogenesis

**Figure 6:**
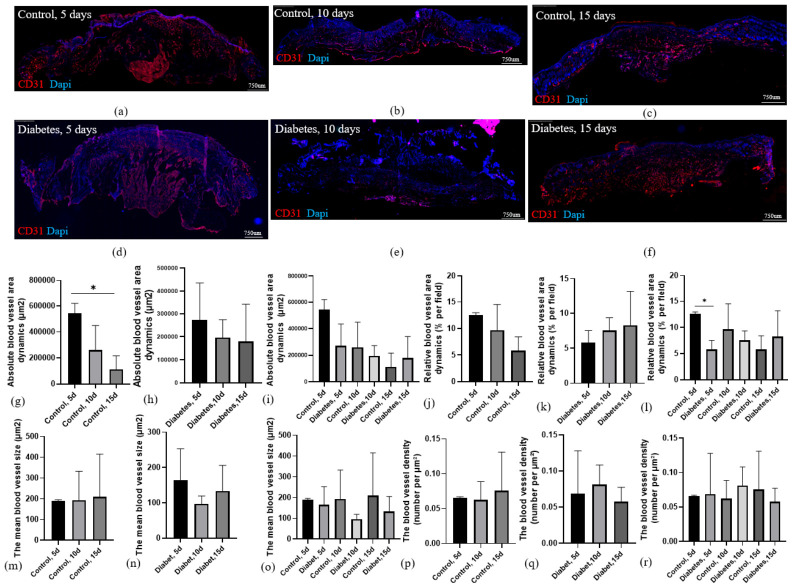
Angiogenesis dynamics in wounds of control and diabetic mice. Immunohistochemical assessment and morphometric analysis of vascularization: (**a**–**f**) CD31 staining of wound sections. Microphotographs showing CD31-positive blood vessels (magnification ×400). Control group: (**a**) day 5, (**b**) day 10, (**c**) day 15; Diabetic group: (**d**) day 5, (**e**) day 10, (**f**) day 15. (**g**–**i**) Absolute blood vessel area. Grouped bar charts showing the total area occupied by vessels in the wound bed: (**g**) comparative analysis between groups; (**h**) control group; (**i**) diabetic group. (**j**–**l**) Relative blood vessel area. Grouped bar charts showing the percentage of vessel area per field: (**j**) comparative analysis between groups; (**k**) control group; (**l**) diabetic group. (**m**–**o**) Blood vessel density. Grouped bar charts representing the number of vessels per µm^2^: (**m**) comparative analysis between groups; (**n**) control group; (**o**) diabetic group. (**p**–**r**) Mean blood vessel size. Grouped bar charts showing the average size of individual vessels: (**p**) comparative analysis between groups; (**q**) control group; (**r**) diabetic group. Statistics: Data are presented as mean ± SD. Ordinary one-way ANOVA with Uncorrected Fisher’s LSD test was used for comparisons. Significance levels: * *p* < 0.05, ** *p* < 0.01.

Angiogenesis, defined as the formation of a new microvascular network within the granulation tissue, was assessed via morphometric analysis of CD31-immunolabeled sections (Figure 6a–f). Quantification within the wound bed encompassed the following parameters: the absolute and relative area (percentage of the microscopic field) occupied by CD31^+^ structures, microvascular density (number of profiles per mm^2^), and mean cross-sectional vessel area.

Relative blood vessel area was calculated in accordance with the formula:

$$A_{rel}=\frac{{\sum S}_{vessel}}{S_{field}}\times 100$$

∑S_vessel_—total area of all CD31^+^ profiles in the field of view, µm^2^.
S_field_—total area of the analyzed field of view (or wound bed), µm^2^.

Microvascular density was evaluated in accordance with the formula:

$$D_{vessel}=\frac{N_{vessel}}{S_{field}}$$

N_vessel_—total number of CD31^+^ profiles (vessels) in the field of view.
S_field_—area of the analyzed tissue region, mm^2^.

Mean cross-sectional vessel area was measured in accordance with the formula:

$$s_{vessel}=\frac{{\sum S}_{vessel}}{N_{vessel}}\times 100$$

∑S_vessel_—total area of all CD31^+^ profiles in the field of view, µm^2^.
N_vessel_—total number of all CD31^+^ profiles in the field of view.

Statistical analysis within the control group demonstrated a significant reduction in absolute vascular area by days 10 and 15 compared with day 5, a finding consistent with the physiological remodeling and maturation of granulation tissue (Figure 6g). In contrast, the diabetic group failed to exhibit this temporal remodeling dynamic (Figure 6h). This finding may suggest a disruption of the normal dynamics of granulation tissue maturation in the proposed diabetic wound model. Quantitative morphometric analysis revealed that on day 5 post-wounding, the absolute vascular area within the granulation tissue was diminished in the diabetic group relative to controls; however, this difference was not statistically significant (Figure 6i).

Assessment of the relative vascular area corroborated these findings, revealing a statistically significant decrease in the Diabetes group on day 5 (Figure 6l). However, this difference was abrogated at subsequent time points. Notably, neither group displayed significant intragroup dynamics in relative vascular area across the day 5, 10, and 15 time points (Figure 6j,k).

Statistical analysis revealed no significant differences in either microvascular density or mean vessel cross-sectional area, both between experimental groups and across the evaluated time points (Figure 6m–r).

Collectively, these findings indicate a delay in angiogenesis during the early inflammatory phase of diabetic wound regeneration, manifested on day 5 as a reduced relative area fraction of the vascular bed within the granulation tissue—pointing to a disrupted stoichiometry between nascent vessels and other extracellular matrix components.

Main results are summarized in Table 2.

## 4. Discussion

In our previous work, we modeled a non-healing wound on an ischemic skin flap. This model was studied on days 5, 7, 14, and 21 and reproduced the key stages of regeneration with a delay in the second phase—the proliferation phase. On day 5, the wound was in the inflammatory stage; from days 7 to 14, in the proliferative stage; and by day 21, in the scar remodeling and re-epithelialization stage. However, this model did not fully reproduce the pathogenetic characteristics of chronic wounds.

It is well established that the key feature of non-healing wounds is a delay in regeneration due to excessive inflammation [1]. Such wounds are characterized by persistent inflammation, elevated proteolytic activity, and reduced extracellular matrix deposition [2]. Healing in chronic wounds proceeds through the same stages as under normal conditions, but with a significant delay specifically in the inflammatory phase [4]. The most important histological biomarker of chronicity is excessive neutrophil infiltration into the wound bed [3].

In the model proposed in the present study, on day 10 (the proliferative phase), the granulation tissue of diabetic mice exhibited a pronounced LNL, whereas the maturing layer dominated in control mice at the same time point. This indicates chronification of inflammation, which underlies the delay in regeneration. Excess neutrophils lead to overproduction of reactive oxygen species (ROS), which damage the extracellular matrix (ECM) and cell membranes, and prematurely reduce cellular functions [3]. ROS activate the expression of pro-inflammatory cytokines (IL-1, IL-6, TNFα), chemokines, and proteolytic enzymes, including matrix metalloproteinases (MMPs) and serine proteases [4]. In addition, neutrophils themselves secrete these enzymes: serine proteases (elastases) cleave growth factors, while MMP collagenases degrade the ECM [3]. Evidence of the formation of sluggish granulation tissue due to ECM degradation in our model is provided by the reduced relief index in the early stages of regeneration of diabetic wounds compared with controls. Furthermore, it is known that excessive MMP-2 and MMP-9 activity in patients with diabetes mellitus leads to excessive ECM degradation, impairing the migration of not only fibroblasts but also endothelial cells, ultimately compromising angiogenesis [14]. Similar disturbances in angiogenesis were observed in our model.

Compared with classical diabetic wound models, the model proposed in the present study offers several advantages. For instance, in a streptozotocin-induced diabetic mouse model with a 6 mm wound, the wound was reported to be fully covered by multilayered epithelium and filled with maturing granulation tissue by day 12, indicating a proliferative stage [10]. In our model, by day 10, the wound was also at the proliferative stage; however, the granulation tissue was less mature, as evidenced by the persistence of a pronounced LNL. Similarly, in db/db mice, a wound of comparable parameters showed even less cellular infiltration than the control [9]. Collectively, these comparisons demonstrate that, unlike classical models, our model more closely recapitulates the pathogenesis of diabetic wounds, which is characterized by delayed inflammation during regeneration.

Thus, we hypothesize that the delayed regeneration in the proposed model is due precisely to the persistence of the inflammatory process, thereby recapitulating the key pathophysiological mechanisms characteristic of chronic human wounds.

A limitation of the present study is that regeneration was described only up to day 15. Whether regeneration is completed and over what time frame remains an open question requiring further investigation. At the same time, the main advantage of the model is not so much the duration of the regenerative process (in mice, even under diabetic conditions, healing rates remain relatively high) but rather the recapitulation of qualitative regeneration defects analogous to those observed in humans.

The developed model is convenient for preclinical testing of various drugs for the treatment of diabetic wounds, including innovative cell-based products and skin equivalents. The square wound shape with an area of 1 × 1 cm is optimal for the transplantation of tissue-engineered constructs, and comprehensively characterized regeneration dynamics allow objective assessment of healing processes at all stages. Therefore, the proposed model can be recommended for preclinical studies of drugs aimed at treating chronic diabetic wounds.

## 5. Conclusions

The integrated analysis of key regenerative parameters demonstrates a significant delay in the regeneration of diabetic wounds relative to acute controls. These parameters include the percentage of re-epithelialization, contraction, epidermal hypertrophy, the relief index, angiogenesis, and the stratification of granulation tissue layers. Collectively, these findings validate that the diabetic wound model established according to our protocol faithfully recapitulates the essential criteria of a chronic, non-healing wound. This model therefore represents a robust experimental system for investigating the pathophysiological mechanisms underlying impaired wound healing in humans, and for preclinical evaluation of potential therapeutic interventions.

## Figures and Tables

**Table 1:** Number of mice per group.

Group	Day	Number of Mice
Control	5	5
Control	10	5
Control	15	6
Diabetes	5	6
Diabetes	10	5
Diabetes	15	5

**Table 2:** Overview of key results.

Parameter	Diabetic vs. Control	Statistical Significance (*p* < 0.05)
Wound area	Comparable	No
Re-epithelization	Delayed closure	No
Epidermal hypertrophy	Persistent marginal epidermal hyperplasia (day 5 and day 10)	Yes
Wound contraction	Delayed	No
Wound closure	Disrupted dynamics	No
Relief index	Impaired granulation tissue remodeling	Yes (day 5)
Angiogenesis	Reduced early vascular area	Yes (day 5)
Stratification of Granulation Tissue Layers	↑ Immature/↓ Mature layers	Yes

## Data Availability

The original contributions presented in this study are included in the article. Further inquiries can be directed to the corresponding authors.

## Abbreviations

The following abbreviations are used in this manuscript:

LNL	Leukocyte-Necrotic Layer
VLL	Vascular Loop layer
VVL	Vertical Vessel Layer
HFL	Horizontal Fibroblast Layer
ML	Maturing Layer
